# 4-(3-Carb­oxy-1-ethyl-6-fluoro-4-oxo-1,4-dihydro-7-quinol­yl)-1-methyl­piper­azin­ium picrate

**DOI:** 10.1107/S1600536810006835

**Published:** 2010-02-27

**Authors:** Hoong-Kun Fun, Madhukar Hemamalini, Divya N. Shetty, B. Narayana, H. S. Yathirajan

**Affiliations:** a X-ray Crystallography Unit, School of Physics, Universiti Sains Malaysia, 11800 USM, Penang, Malaysia; b Department of Studies in Chemistry, Mangalore University, Mangalagangotri 574 199, India; c Department of Studies in Chemistry, University of Mysore, Manasagangotri, Mysore 570 006, India

## Abstract

The pefloxacinium cation of the title salt, C_17_H_21_FN_3_O_3_^+^·C_6_H_2_N_3_O_7_^−^, is composed of an essentially planar quinoline ring system [maximum deviation = 0.021 (2) Å] and a piperazine ring, which adopts a chair conformation. In the picrate anion, the two O atoms of one of the *o*-NO_2_ groups are disordered over two positions, with an occupancy ratio of 0.56 (4):0.44 (4). In the crystal structure, cations and anions are connected by inter­molecular N—H⋯O, O—H⋯O, C—H⋯O and C—H⋯F hydrogen bonds, forming a three-dimensional network. In addition, π–π inter­actions between the pyridine rings and between the benzene rings of the anions, with centroid–centroid distances of 3.6103 (12) and 3.5298 (11) Å, respectively, are observed.

## Related literature

For background to the biological activity, pharmacokinetic properties and therapeutic use of pefloxacin, a synthetic chemotherapeutic agent used to treat severe bacterial infections, see: Mizuki *et al.* (1996 ▶); Gonzalez & Henwood (1989 ▶); Tripathi (1995 ▶); Ross & Riley (1990 ▶); Burkhardt *et al.* (1997 ▶). For the silver(I), manganese(II) and cobalt(II) derivatives of the pefloxacin anion, see: Baenziger *et al.* (1986 ▶); An, Huang & Qi (2007 ▶); An, Qi & Huang (2007 ▶). For related structures, see; An & Liang (2008 ▶); Florence *et al.* (2000 ▶); Hu & Yu (2005 ▶); Parvez *et al.* (2000 ▶). For hydrogen-bonding motifs see: Bernstein *et al.* (1995 ▶). For ring conformations, see: Cremer & Pople (1975 ▶).
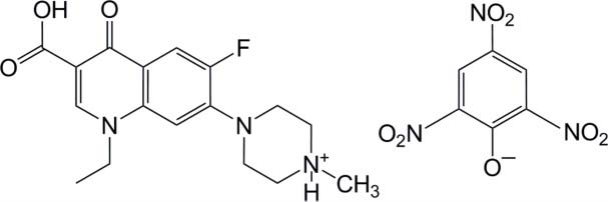


## Experimental

### 

#### Crystal data

C_17_H_21_FN_3_O_3_^+^·C_6_H_2_N_3_O_7_^−^
*M* *_r_* = 562.47
Triclinic, 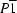

*a* = 7.2645 (1) Å
*b* = 9.1987 (2) Å
*c* = 20.2253 (4) Å
α = 77.116 (1)°
β = 81.315 (1)°
γ = 67.124 (1)°
*V* = 1210.77 (4) Å^3^
*Z* = 2
Mo *K*α radiation
μ = 0.13 mm^−1^
*T* = 296 K
0.36 × 0.13 × 0.13 mm

#### Data collection

Bruker SMART APEXII CCD area-detector diffractometer
Absorption correction: multi-scan (*SADABS*; Bruker, 2009 ▶) *T*_min_ = 0.955, *T*_max_ = 0.984
23708 measured reflections
5523 independent reflections
3682 reflections with *I* > 2s(*I*)
*R*_int_ = 0.039

#### Refinement

*R*[*F* ^2^ > 2σ(*F* ^2^)] = 0.047
*wR*(*F* ^2^) = 0.123
*S* = 1.03
5523 reflections
472 parameters
All H-atom parameters refined
Δρ_max_ = 0.21 e Å^−3^
Δρ_min_ = −0.22 e Å^−3^

### 

Data collection: *APEX2* (Bruker, 2009 ▶); cell refinement: *SAINT* (Bruker, 2009 ▶); data reduction: *SAINT*; program(s) used to solve structure: *SHELXTL* (Sheldrick, 2008 ▶); program(s) used to refine structure: *SHELXTL*; molecular graphics: *SHELXTL*; software used to prepare material for publication: *SHELXTL* and *PLATON* (Spek, 2009 ▶).

## Supplementary Material

Crystal structure: contains datablocks global, I. DOI: 10.1107/S1600536810006835/tk2633sup1.cif

Structure factors: contains datablocks I. DOI: 10.1107/S1600536810006835/tk2633Isup2.hkl

Additional supplementary materials:  crystallographic information; 3D view; checkCIF report

## Figures and Tables

**Table 1:** Hydrogen-bond geometry (Å, °)

*D*—H⋯*A*	*D*—H	H⋯*A*	*D*⋯*A*	*D*—H⋯*A*
O9—H1*O*9⋯O10	0.96 (3)	1.62 (3)	2.519 (2)	155 (3)
N6—H1*N*6⋯O1^i^	0.91 (2)	1.84 (2)	2.701 (2)	159 (2)
N6—H1*N*6⋯O7^i^	0.91 (2)	2.422 (19)	2.987 (2)	120.6 (15)
C6—H6⋯O3	0.878 (19)	2.38 (2)	2.706 (16)	102.3 (14)
C11—H11⋯F1^ii^	0.933 (19)	2.46 (2)	3.209 (2)	137.3 (18)
C16—H16*A*⋯F1	0.97 (2)	2.18 (2)	2.846 (2)	124.6 (15)
C17—H17*A*⋯O8^iii^	0.928 (19)	2.582 (19)	3.430 (3)	152.2 (14)
C17—H17*B*⋯O4^iv^	1.02 (2)	2.55 (2)	3.407 (3)	141.2 (16)
C18—H18*A*⋯O3^v^	0.940 (19)	2.47 (2)	3.241 (16)	139.2 (18)
C19—H19*B*⋯O5^vi^	0.990 (17)	2.566 (17)	3.300 (2)	131.0 (13)
C20—H20*A*⋯O7^i^	0.97 (2)	2.51 (2)	3.079 (3)	118.0 (18)
C20—H20*C*⋯O4^iv^	1.01 (3)	2.39 (3)	3.245 (3)	141.8 (19)
C21—H21*B*⋯O8^vii^	0.90 (3)	2.53 (3)	3.415 (3)	169 (2)

Symmetry codes: (i) 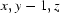; (ii) 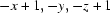; (iii) 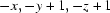; (iv) 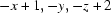; (v) 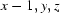; (vi) 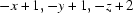; (vii) 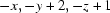.

**Fig. 1.**
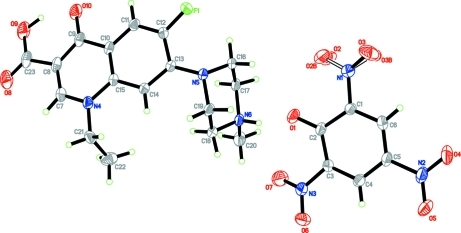
The asymmetric unit of the title compound, showing 50% probability displacement ellipsoids and the atom-numbering scheme.

**Fig. 2.**
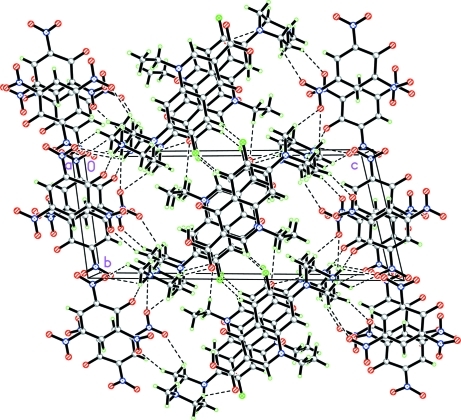
The crystal packing of the title compound, showing hydrogen-bonded (dashed lines) network.

## supplementary crystallographic information

### Comment

Pefloxacin is a synthetic chemotherapeutic agent used to treat severe and life-threatening bacterial infections (Mizuki *et al.*, 1996). A review of its anti-bacterial activity, pharmacokinetic properties and therapeutic use is available (Gonzalez & Henwood, 1989). Pefloxacin is commonly referred to as a fluoroquinolone (or quinolone) drug and is a member of the fluoroquinolone class of anti-bacterials. It is an analog of norfloxacin and is a synthetic fluoroquinolone, belonging to the third generation of quinolones. As an antibacterial drug, it is highly effective against both Gram-negative and Gram-positive pathogens that are resistant to other anti-bacterials (Tripathi, 1995; Ross *et al.*, 1990). Pefloxacin is well known to be associated with high incidence of arthropathy in humans because the drug affects articular cartilage and the epiphyseal growth plate (Burkhardt *et al.*, 1997). The silver(I), manganese(II) and cobalt(II) derivatives of the pefloxacin anion have been reported (Baenziger *et al.*, 1986; An, Huang, & Qi, 2007; An, Qi & Huang, 2007). The crystal structures of silver pefloxacin (Baenziger *et al.*, 1986), pefloxacinium methane sulfonate 0.10-hydrate (Parvez *et al.*, 2000), norfloxacin dihydrate (Florence *et al.*, 2000), norfloxacin picrate (Hu & Yu, 2005), 1-ethyl-6-fluoro-7-(4-methylpiperazin-4-ium-1-yl) -4-oxo-1,4-dihydroquinoline-3-carboxylate hexahydrate (An & Liang, 2008) have been reported. In view of the importance of pefloxacin, this paper reports the crystal structure of the title compound.

The asymmetric unit of the title compound (Fig. 1), contains a protonated pefloxacinium cation and a picrate anion. The cation is composed of an essentially planar quinoline ring system [maximum deviation 0.021 (2) Å]. The six-membered piperazinyl ring adopts a chair conformation with puckering parameters (Cremer & Pople, 1975) Q = 0.5621 (2) Å , Θ = 174.05 (18)° and φ = 169 (2)°. In the picrate anion, atoms O2 and O3 are disordered over two positions, with occupancy ratio of 0.56 (4):0.44 (4). The phenolate oxygen atoms are bent slightly away from the mean plane of the benzene ring (torsion angle O1—C2—C3—C4 = -177.76 (18)°).

In the crystal structure (Fig. 2), the picrate anion interacts with the cations through bifurcated N6—H1N6···O1 and N6—H1N6···O7 hydrogen bonds, forming an *R*_1_^2^(6) (Bernstein *et al.*, 1995) ring motif. There is an intramolecular O9—H1O9···O10 hydrogen bond between the carbonyl and carboxyl groups in the cation, which generates an *S*(6) ring motif. The crystal structure is further stabilized by several weak C—H···O and C—H···F interactions (Table 1), forming a three-dimensional network. Also, π–π interactions between pyridine rings, and between benzene rings of anions/anions, with centroid-to-centroid distances = 3.6103 (12) Å and 3.5298 (11) Å, respectively, are observed.

### Experimental

Each of the pefloxacin (3.33 g, 0.01 mol) and picric acid (2.29 g, 0.01 mol) were individually dissolved in water (60 ml). The solutions were mixed and 5 M HCl (2 ml) was added with stirring for a few minutes. The product formed was filtered and dried. Yellow crystals of pefloxacinium picrate were obtained by slow evaporation in DMF (m.p.: 515 K).

### Refinement

All the H atoms were located in a difference Fourier map and allowed to refine freely [N–H = 0.905 (19) Å, O—H = 0.96 (3) Å, C–H = 0.88 (19)–1.01 (3) Å]. In the picrate anion, atoms O2 and O3 are disordered over two positions, with an occupancy ratio of 0.56 (4):0.44 (4).

### Crystal data

**Table:**

C_17_H_21_FN_3_O_3_^+^·C_6_H_2_N_3_O_7_^−^	*Z* = 2
*M**_r_* = 562.47	*F*(000) = 584
Triclinic, *P*1	*D*_x_ = 1.543 Mg m^−^^3^
Hall symbol: -P 1	Mo *K*α radiation, λ = 0.71073 Å
*a* = 7.2645 (1) Å	Cell parameters from 4949 reflections
*b* = 9.1987 (2) Å	θ = 2.5–31.8°
*c* = 20.2253 (4) Å	µ = 0.13 mm^−^^1^
α = 77.116 (1)°	*T* = 296 K
β = 81.315 (1)°	Block, yellow
γ = 67.124 (1)°	0.36 × 0.13 × 0.13 mm
*V* = 1210.77 (4) Å^3^	

### Data collection

**Table:**

Bruker SMART APEXII CCD area-detector diffractometer	5523 independent reflections
Radiation source: fine-focus sealed tube	3682 reflections with *I* > 2s(*I*)
graphite	*R*_int_ = 0.039
φ and ω scans	θ_max_ = 27.5°, θ_min_ = 2.1°
Absorption correction: multi-scan (*SADABS*; Bruker, 2009)	*h* = −9→9
*T*_min_ = 0.955, *T*_max_ = 0.984	*k* = −11→11
23708 measured reflections	*l* = −26→26

### Refinement

**Table:**

Refinement on *F*^2^	Primary atom site location: structure-invariant direct methods
Least-squares matrix: full	Secondary atom site location: difference Fourier map
*R*[*F*^2^ > 2σ(*F*^2^)] = 0.047	Hydrogen site location: inferred from neighbouring sites
*wR*(*F*^2^) = 0.123	All H-atom parameters refined
*S* = 1.03	*w* = 1/[σ^2^(*F*_o_^2^) + (0.0539*P*)^2^ + 0.1877*P*] where *P* = (*F*_o_^2^ + 2*F*_c_^2^)/3
5523 reflections	(Δ/σ)_max_ = 0.001
472 parameters	Δρ_max_ = 0.21 e Å^−^^3^
0 restraints	Δρ_min_ = −0.21 e Å^−^^3^

### Special details

**Table:**

Geometry. All esds (except the esd in the dihedral angle between two l.s. planes) are estimated using the full covariance matrix. The cell esds are taken into account individually in the estimation of esds in distances, angles and torsion angles; correlations between esds in cell parameters are only used when they are defined by crystal symmetry. An approximate (isotropic) treatment of cell esds is used for estimating esds involving l.s. planes.
Refinement. Refinement of F^2^ against ALL reflections. The weighted R-factor wR and goodness of fit S are based on F^2^, conventional R-factors R are based on F, with F set to zero for negative F^2^. The threshold expression of F^2^ > 2sigma(F^2^) is used only for calculating R-factors(gt) etc. and is not relevant to the choice of reflections for refinement. R-factors based on F^2^ are statistically about twice as large as those based on F, and R- factors based on ALL data will be even larger.

### Fractional atomic coordinates and isotropic or equivalent isotropic displacement parameters (Å^2^)

**Table:**

	*x*	*y*	*z*	*U*_iso_*/*U*_eq_	Occ. (<1)
O1	0.4979 (2)	0.79508 (17)	0.88294 (6)	0.0614 (4)	
O2	0.7151 (13)	0.5178 (13)	0.8445 (5)	0.067 (2)	0.56 (4)
O3	0.9703 (16)	0.3754 (18)	0.9011 (8)	0.075 (3)	0.56 (4)
O2B	0.768 (5)	0.546 (3)	0.8376 (7)	0.120 (6)	0.44 (4)
O3B	0.934 (4)	0.357 (3)	0.9074 (10)	0.103 (5)	0.44 (4)
O4	0.8735 (3)	0.32881 (19)	1.14260 (8)	0.0788 (5)	
O5	0.6553 (3)	0.5215 (2)	1.18879 (7)	0.0730 (5)	
O6	0.2844 (2)	1.00907 (18)	1.04797 (8)	0.0663 (4)	
O7	0.2049 (3)	0.9961 (2)	0.95255 (9)	0.0994 (7)	
N1	0.8047 (2)	0.4832 (2)	0.89513 (8)	0.0473 (4)	
N2	0.7383 (3)	0.4603 (2)	1.13878 (9)	0.0543 (4)	
N3	0.3077 (3)	0.9389 (2)	1.00085 (9)	0.0531 (4)	
C1	0.7049 (2)	0.5634 (2)	0.95257 (8)	0.0367 (4)	
C2	0.5487 (2)	0.7206 (2)	0.94034 (8)	0.0388 (4)	
C3	0.4645 (3)	0.7803 (2)	1.00324 (9)	0.0391 (4)	
C4	0.5270 (3)	0.6985 (2)	1.06618 (10)	0.0422 (4)	
C5	0.6760 (3)	0.5484 (2)	1.07199 (9)	0.0425 (4)	
C6	0.7669 (3)	0.4806 (2)	1.01542 (9)	0.0404 (4)	
F1	0.63677 (18)	−0.03326 (12)	0.57808 (5)	0.0561 (3)	
O8	−0.0325 (3)	0.9171 (2)	0.40639 (8)	0.0782 (5)	
O9	0.0924 (3)	0.7207 (2)	0.34701 (8)	0.0711 (5)	
O10	0.2917 (2)	0.43985 (18)	0.40332 (7)	0.0611 (4)	
N4	0.2108 (2)	0.60713 (17)	0.58140 (7)	0.0395 (3)	
N5	0.5335 (2)	0.06173 (16)	0.70402 (7)	0.0355 (3)	
N6	0.2153 (2)	0.01715 (17)	0.80261 (7)	0.0381 (3)	
C7	0.1335 (3)	0.7054 (2)	0.52424 (10)	0.0452 (5)	
C8	0.1526 (3)	0.6553 (2)	0.46406 (9)	0.0456 (5)	
C9	0.2625 (3)	0.4915 (2)	0.45840 (9)	0.0440 (5)	
C10	0.3409 (3)	0.3855 (2)	0.52080 (8)	0.0375 (4)	
C11	0.4521 (3)	0.2212 (2)	0.52173 (9)	0.0419 (4)	
C12	0.5179 (3)	0.1222 (2)	0.58059 (9)	0.0386 (4)	
C13	0.4736 (2)	0.1723 (2)	0.64432 (8)	0.0329 (4)	
C14	0.3771 (2)	0.3363 (2)	0.64216 (9)	0.0341 (4)	
C15	0.3112 (2)	0.4432 (2)	0.58177 (8)	0.0343 (4)	
C16	0.4921 (3)	−0.0875 (2)	0.71661 (10)	0.0403 (4)	
C17	0.2763 (3)	−0.0593 (2)	0.74081 (9)	0.0403 (4)	
C18	0.2690 (3)	0.1625 (2)	0.79291 (10)	0.0409 (4)	
C19	0.4868 (3)	0.1236 (2)	0.76759 (9)	0.0386 (4)	
C20	−0.0031 (3)	0.0581 (3)	0.82167 (14)	0.0571 (6)	
C21	0.1823 (3)	0.6765 (3)	0.64328 (11)	0.0493 (5)	
C22	0.0028 (4)	0.6633 (4)	0.68827 (13)	0.0664 (7)	
C23	0.0618 (3)	0.7773 (3)	0.40435 (11)	0.0563 (6)	
H4	0.468 (3)	0.743 (2)	1.1031 (11)	0.055 (6)*	
H6	0.864 (3)	0.386 (2)	1.0177 (9)	0.046 (5)*	
H7	0.066 (3)	0.813 (3)	0.5297 (10)	0.055 (6)*	
H11	0.485 (3)	0.181 (2)	0.4813 (10)	0.047 (5)*	
H14	0.349 (2)	0.379 (2)	0.6821 (9)	0.036 (5)*	
H16A	0.526 (3)	−0.136 (2)	0.6761 (11)	0.057 (6)*	
H16B	0.583 (3)	−0.163 (2)	0.7505 (10)	0.053 (5)*	
H17A	0.193 (3)	0.012 (2)	0.7078 (9)	0.044 (5)*	
H17B	0.251 (3)	−0.164 (3)	0.7550 (10)	0.059 (6)*	
H18A	0.245 (3)	0.196 (2)	0.8351 (10)	0.043 (5)*	
H18B	0.182 (3)	0.241 (2)	0.7603 (10)	0.045 (5)*	
H19A	0.569 (3)	0.039 (2)	0.8001 (9)	0.040 (5)*	
H19B	0.521 (2)	0.220 (2)	0.7621 (9)	0.040 (5)*	
H20A	−0.038 (3)	0.107 (3)	0.8617 (12)	0.072 (7)*	
H20B	−0.079 (4)	0.131 (3)	0.7848 (13)	0.081 (8)*	
H20C	−0.033 (3)	−0.043 (3)	0.8318 (12)	0.080 (8)*	
H21A	0.303 (3)	0.623 (2)	0.6670 (9)	0.046 (5)*	
H21B	0.161 (3)	0.781 (3)	0.6285 (10)	0.055 (6)*	
H22A	−0.106 (4)	0.710 (3)	0.6634 (14)	0.096 (9)*	
H22B	0.022 (4)	0.552 (4)	0.7065 (13)	0.092 (9)*	
H22C	−0.013 (4)	0.715 (3)	0.7274 (15)	0.103 (9)*	
H1O9	0.179 (4)	0.610 (4)	0.3556 (15)	0.110 (11)*	
H1N6	0.284 (3)	−0.055 (2)	0.8372 (10)	0.044 (5)*	

### Atomic displacement parameters (Å^2^)

**Table:**

	*U*^11^	*U*^22^	*U*^33^	*U*^12^	*U*^13^	*U*^23^
O1	0.0640 (9)	0.0613 (9)	0.0329 (7)	−0.0004 (7)	−0.0099 (6)	0.0052 (6)
O2	0.075 (3)	0.091 (4)	0.032 (3)	−0.022 (3)	−0.008 (2)	−0.019 (3)
O3	0.046 (3)	0.075 (4)	0.094 (7)	−0.001 (3)	−0.005 (3)	−0.037 (4)
O2B	0.146 (11)	0.093 (7)	0.041 (3)	0.026 (7)	0.016 (6)	0.001 (4)
O3B	0.114 (10)	0.072 (7)	0.058 (5)	0.034 (7)	0.006 (7)	−0.015 (4)
O4	0.1096 (14)	0.0488 (10)	0.0600 (10)	−0.0117 (9)	−0.0349 (9)	0.0128 (8)
O5	0.1082 (13)	0.0755 (11)	0.0342 (8)	−0.0327 (10)	−0.0172 (8)	−0.0017 (8)
O6	0.0818 (10)	0.0514 (9)	0.0601 (10)	−0.0210 (8)	0.0176 (8)	−0.0208 (8)
O7	0.0998 (13)	0.0821 (13)	0.0596 (11)	0.0313 (10)	−0.0219 (10)	−0.0093 (9)
N1	0.0471 (9)	0.0478 (11)	0.0434 (10)	−0.0148 (8)	0.0000 (8)	−0.0083 (8)
N2	0.0789 (12)	0.0483 (11)	0.0388 (10)	−0.0286 (9)	−0.0205 (9)	0.0067 (8)
N3	0.0613 (10)	0.0434 (10)	0.0418 (10)	−0.0125 (8)	0.0051 (8)	−0.0009 (8)
C1	0.0402 (9)	0.0377 (10)	0.0325 (9)	−0.0152 (7)	−0.0031 (7)	−0.0047 (8)
C2	0.0416 (9)	0.0395 (10)	0.0310 (9)	−0.0138 (8)	−0.0066 (7)	0.0024 (8)
C3	0.0439 (9)	0.0336 (10)	0.0362 (10)	−0.0135 (7)	−0.0027 (7)	−0.0010 (8)
C4	0.0556 (11)	0.0425 (11)	0.0330 (10)	−0.0240 (9)	−0.0026 (8)	−0.0049 (8)
C5	0.0565 (11)	0.0398 (11)	0.0333 (10)	−0.0226 (9)	−0.0138 (8)	0.0050 (8)
C6	0.0438 (10)	0.0316 (10)	0.0443 (11)	−0.0131 (8)	−0.0118 (8)	0.0006 (8)
F1	0.0842 (8)	0.0348 (6)	0.0393 (6)	−0.0115 (5)	−0.0012 (5)	−0.0083 (5)
O8	0.0905 (12)	0.0572 (11)	0.0693 (11)	−0.0164 (9)	−0.0349 (9)	0.0226 (8)
O9	0.0922 (12)	0.0720 (12)	0.0470 (9)	−0.0327 (10)	−0.0345 (8)	0.0176 (8)
O10	0.0940 (11)	0.0621 (10)	0.0345 (8)	−0.0367 (8)	−0.0223 (7)	0.0031 (7)
N4	0.0437 (8)	0.0334 (8)	0.0361 (8)	−0.0114 (6)	−0.0087 (6)	0.0029 (6)
N5	0.0403 (7)	0.0311 (8)	0.0285 (7)	−0.0093 (6)	−0.0023 (6)	0.0002 (6)
N6	0.0377 (7)	0.0351 (8)	0.0320 (8)	−0.0092 (6)	−0.0041 (6)	0.0057 (7)
C7	0.0468 (10)	0.0387 (11)	0.0453 (11)	−0.0154 (9)	−0.0107 (8)	0.0066 (9)
C8	0.0454 (10)	0.0489 (12)	0.0415 (11)	−0.0233 (9)	−0.0161 (8)	0.0133 (9)
C9	0.0529 (11)	0.0516 (12)	0.0352 (10)	−0.0310 (9)	−0.0149 (8)	0.0066 (9)
C10	0.0455 (9)	0.0406 (10)	0.0299 (9)	−0.0230 (8)	−0.0085 (7)	0.0038 (7)
C11	0.0595 (11)	0.0435 (11)	0.0293 (9)	−0.0265 (9)	−0.0054 (8)	−0.0045 (8)
C12	0.0490 (10)	0.0306 (10)	0.0352 (10)	−0.0154 (8)	−0.0019 (8)	−0.0033 (7)
C13	0.0350 (8)	0.0342 (9)	0.0281 (9)	−0.0141 (7)	−0.0029 (7)	0.0002 (7)
C14	0.0387 (9)	0.0334 (10)	0.0287 (9)	−0.0129 (7)	−0.0033 (7)	−0.0030 (7)
C15	0.0360 (8)	0.0324 (9)	0.0332 (9)	−0.0144 (7)	−0.0063 (7)	0.0025 (7)
C16	0.0508 (10)	0.0286 (10)	0.0322 (10)	−0.0089 (8)	−0.0008 (8)	0.0012 (8)
C17	0.0506 (10)	0.0344 (10)	0.0328 (10)	−0.0154 (8)	−0.0088 (8)	0.0035 (8)
C18	0.0506 (10)	0.0355 (10)	0.0314 (10)	−0.0132 (8)	0.0014 (8)	−0.0033 (8)
C19	0.0456 (10)	0.0404 (11)	0.0277 (9)	−0.0156 (8)	−0.0083 (7)	0.0013 (8)
C20	0.0404 (10)	0.0558 (14)	0.0611 (15)	−0.0133 (10)	0.0024 (10)	0.0048 (12)
C21	0.0618 (13)	0.0315 (11)	0.0475 (12)	−0.0074 (9)	−0.0142 (10)	−0.0037 (9)
C22	0.0521 (13)	0.080 (2)	0.0512 (14)	−0.0029 (12)	−0.0062 (11)	−0.0177 (14)
C23	0.0603 (12)	0.0582 (14)	0.0483 (13)	−0.0283 (11)	−0.0245 (10)	0.0201 (11)

### Geometric parameters (Å, °)

**Table:**

O1—C2	1.2375 (19)	N6—H1N6	0.905 (19)
O2—N1	1.212 (8)	C7—C8	1.365 (3)
O3—N1	1.228 (13)	C7—H7	0.94 (2)
O2B—N1	1.196 (13)	C8—C9	1.426 (3)
O3B—N1	1.177 (17)	C8—C23	1.488 (3)
O4—N2	1.221 (2)	C9—C10	1.451 (2)
O5—N2	1.229 (2)	C10—C15	1.404 (2)
O6—N3	1.222 (2)	C10—C11	1.407 (3)
O7—N3	1.214 (2)	C11—C12	1.351 (2)
N1—C1	1.460 (2)	C11—H11	0.932 (19)
N2—C5	1.448 (2)	C12—C13	1.415 (2)
N3—C3	1.457 (2)	C13—C14	1.388 (2)
C1—C6	1.368 (2)	C14—C15	1.401 (2)
C1—C2	1.446 (2)	C14—H14	0.936 (17)
C2—C3	1.452 (2)	C16—C17	1.508 (3)
C3—C4	1.369 (2)	C16—H16A	0.97 (2)
C4—C5	1.377 (3)	C16—H16B	0.98 (2)
C4—H4	0.90 (2)	C17—H17A	0.929 (19)
C5—C6	1.380 (3)	C17—H17B	1.02 (2)
C6—H6	0.882 (19)	C18—C19	1.514 (3)
F1—C12	1.3609 (19)	C18—H18A	0.942 (19)
O8—C23	1.206 (3)	C18—H18B	0.956 (19)
O9—C23	1.329 (3)	C19—H19A	0.959 (18)
O9—H1O9	0.96 (3)	C19—H19B	0.994 (18)
O10—C9	1.264 (2)	C20—H20A	0.97 (2)
N4—C7	1.343 (2)	C20—H20B	0.96 (3)
N4—C15	1.396 (2)	C20—H20C	1.01 (3)
N4—C21	1.480 (2)	C21—C22	1.502 (3)
N5—C13	1.396 (2)	C21—H21A	0.964 (19)
N5—C19	1.462 (2)	C21—H21B	0.90 (2)
N5—C16	1.477 (2)	C22—H22A	0.91 (3)
N6—C20	1.491 (2)	C22—H22B	0.97 (3)
N6—C17	1.496 (2)	C22—H22C	0.98 (3)
N6—C18	1.498 (2)		
			
O3B—N1—O2B	120.7 (12)	C12—C11—H11	119.8 (12)
O3B—N1—O2	119.9 (11)	C10—C11—H11	119.8 (12)
O2B—N1—O2	25.4 (19)	C11—C12—F1	118.09 (16)
O3B—N1—O3	17.2 (18)	C11—C12—C13	123.53 (16)
O2B—N1—O3	114.2 (11)	F1—C12—C13	118.36 (14)
O2—N1—O3	121.9 (8)	C14—C13—N5	123.69 (15)
O3B—N1—C1	116.8 (10)	C14—C13—C12	115.50 (15)
O2B—N1—C1	122.4 (7)	N5—C13—C12	120.75 (15)
O2—N1—C1	118.6 (5)	C13—C14—C15	122.18 (16)
O3—N1—C1	119.3 (7)	C13—C14—H14	120.3 (11)
O4—N2—O5	122.98 (17)	C15—C14—H14	117.5 (11)
O4—N2—C5	118.14 (18)	N4—C15—C14	120.91 (15)
O5—N2—C5	118.87 (17)	N4—C15—C10	118.98 (14)
O7—N3—O6	122.52 (18)	C14—C15—C10	120.08 (16)
O7—N3—C3	119.07 (17)	N5—C16—C17	111.86 (15)
O6—N3—C3	118.41 (17)	N5—C16—H16A	111.3 (12)
C6—C1—C2	124.48 (16)	C17—C16—H16A	109.6 (12)
C6—C1—N1	116.28 (16)	N5—C16—H16B	106.4 (11)
C2—C1—N1	119.24 (15)	C17—C16—H16B	111.3 (11)
O1—C2—C1	123.51 (16)	H16A—C16—H16B	106.2 (16)
O1—C2—C3	124.90 (16)	N6—C17—C16	111.76 (15)
C1—C2—C3	111.58 (14)	N6—C17—H17A	105.8 (11)
C4—C3—C2	124.16 (16)	C16—C17—H17A	110.2 (11)
C4—C3—N3	116.38 (16)	N6—C17—H17B	105.1 (11)
C2—C3—N3	119.44 (15)	C16—C17—H17B	112.0 (11)
C3—C4—C5	119.36 (18)	H17A—C17—H17B	111.7 (16)
C3—C4—H4	119.3 (13)	N6—C18—C19	110.49 (15)
C5—C4—H4	121.4 (13)	N6—C18—H18A	107.9 (11)
C4—C5—C6	121.23 (16)	C19—C18—H18A	110.3 (11)
C4—C5—N2	119.28 (18)	N6—C18—H18B	105.3 (11)
C6—C5—N2	119.49 (17)	C19—C18—H18B	111.5 (11)
C1—C6—C5	119.18 (17)	H18A—C18—H18B	111.3 (16)
C1—C6—H6	117.8 (12)	N5—C19—C18	112.19 (14)
C5—C6—H6	123.0 (12)	N5—C19—H19A	105.6 (10)
C23—O9—H1O9	108.0 (18)	C18—C19—H19A	108.9 (10)
C7—N4—C15	119.85 (16)	N5—C19—H19B	110.7 (10)
C7—N4—C21	118.47 (16)	C18—C19—H19B	109.8 (10)
C15—N4—C21	121.66 (14)	H19A—C19—H19B	109.6 (14)
C13—N5—C19	117.44 (14)	N6—C20—H20A	108.9 (14)
C13—N5—C16	118.83 (14)	N6—C20—H20B	109.9 (14)
C19—N5—C16	108.08 (14)	H20A—C20—H20B	110 (2)
C20—N6—C17	110.80 (17)	N6—C20—H20C	109.0 (14)
C20—N6—C18	110.72 (16)	H20A—C20—H20C	110 (2)
C17—N6—C18	111.68 (14)	H20B—C20—H20C	109.3 (19)
C20—N6—H1N6	108.7 (11)	N4—C21—C22	112.15 (19)
C17—N6—H1N6	107.7 (11)	N4—C21—H21A	108.2 (11)
C18—N6—H1N6	107.1 (12)	C22—C21—H21A	111.4 (11)
N4—C7—C8	123.61 (19)	N4—C21—H21B	105.7 (13)
N4—C7—H7	113.1 (12)	C22—C21—H21B	108.4 (13)
C8—C7—H7	123.3 (12)	H21A—C21—H21B	110.9 (17)
C7—C8—C9	120.63 (16)	C21—C22—H22A	108.4 (17)
C7—C8—C23	118.05 (19)	C21—C22—H22B	110.9 (15)
C9—C8—C23	121.28 (19)	H22A—C22—H22B	110 (2)
O10—C9—C8	123.29 (16)	C21—C22—H22C	109.2 (17)
O10—C9—C10	121.36 (18)	H22A—C22—H22C	112 (2)
C8—C9—C10	115.36 (17)	H22B—C22—H22C	107 (2)
C15—C10—C11	117.85 (15)	O8—C23—O9	121.43 (19)
C15—C10—C9	121.42 (17)	O8—C23—C8	123.9 (2)
C11—C10—C9	120.73 (16)	O9—C23—C8	114.6 (2)
C12—C11—C10	120.33 (17)		
			
O3B—N1—C1—C6	−2.1 (18)	O10—C9—C10—C11	−0.1 (3)
O2B—N1—C1—C6	−177 (2)	C8—C9—C10—C11	−179.94 (16)
O2—N1—C1—C6	153.5 (6)	C15—C10—C11—C12	3.5 (3)
O3—N1—C1—C6	−21.4 (8)	C9—C10—C11—C12	−177.41 (16)
O3B—N1—C1—C2	178.6 (18)	C10—C11—C12—F1	−175.41 (15)
O2B—N1—C1—C2	3(2)	C10—C11—C12—C13	3.1 (3)
O2—N1—C1—C2	−25.9 (6)	C19—N5—C13—C14	1.4 (2)
O3—N1—C1—C2	159.2 (7)	C16—N5—C13—C14	134.68 (17)
C6—C1—C2—O1	178.26 (18)	C19—N5—C13—C12	178.36 (15)
N1—C1—C2—O1	−2.5 (3)	C16—N5—C13—C12	−48.4 (2)
C6—C1—C2—C3	−0.2 (2)	C11—C12—C13—C14	−7.6 (2)
N1—C1—C2—C3	179.05 (15)	F1—C12—C13—C14	170.87 (14)
O1—C2—C3—C4	−177.76 (18)	C11—C12—C13—N5	175.20 (16)
C1—C2—C3—C4	0.7 (2)	F1—C12—C13—N5	−6.3 (2)
O1—C2—C3—N3	0.3 (3)	N5—C13—C14—C15	−177.14 (15)
C1—C2—C3—N3	178.69 (15)	C12—C13—C14—C15	5.8 (2)
O7—N3—C3—C4	−157.8 (2)	C7—N4—C15—C14	−174.14 (15)
O6—N3—C3—C4	21.9 (2)	C21—N4—C15—C14	4.4 (2)
O7—N3—C3—C2	24.0 (3)	C7—N4—C15—C10	4.2 (2)
O6—N3—C3—C2	−156.28 (17)	C21—N4—C15—C10	−177.34 (16)
C2—C3—C4—C5	−1.4 (3)	C13—C14—C15—N4	178.65 (15)
N3—C3—C4—C5	−179.47 (16)	C13—C14—C15—C10	0.4 (2)
C3—C4—C5—C6	1.6 (3)	C11—C10—C15—N4	176.55 (15)
C3—C4—C5—N2	−178.45 (16)	C9—C10—C15—N4	−2.6 (2)
O4—N2—C5—C4	−178.56 (18)	C11—C10—C15—C14	−5.1 (2)
O5—N2—C5—C4	0.5 (3)	C9—C10—C15—C14	175.75 (15)
O4—N2—C5—C6	1.4 (3)	C13—N5—C16—C17	−77.71 (19)
O5—N2—C5—C6	−179.54 (17)	C19—N5—C16—C17	59.43 (18)
C2—C1—C6—C5	0.5 (3)	C20—N6—C17—C16	174.57 (16)
N1—C1—C6—C5	−178.80 (15)	C18—N6—C17—C16	50.64 (19)
C4—C5—C6—C1	−1.2 (3)	N5—C16—C17—N6	−55.40 (19)
N2—C5—C6—C1	178.90 (16)	C20—N6—C18—C19	−175.03 (17)
C15—N4—C7—C8	−2.3 (3)	C17—N6—C18—C19	−51.05 (19)
C21—N4—C7—C8	179.16 (17)	C13—N5—C19—C18	77.00 (19)
N4—C7—C8—C9	−1.3 (3)	C16—N5—C19—C18	−60.83 (18)
N4—C7—C8—C23	−179.19 (17)	N6—C18—C19—N5	57.51 (19)
C7—C8—C9—O10	−177.07 (17)	C7—N4—C21—C22	94.7 (2)
C23—C8—C9—O10	0.7 (3)	C15—N4—C21—C22	−83.8 (2)
C7—C8—C9—C10	2.8 (2)	C7—C8—C23—O8	−2.2 (3)
C23—C8—C9—C10	−179.41 (16)	C9—C8—C23—O8	179.97 (19)
O10—C9—C10—C15	179.02 (15)	C7—C8—C23—O9	177.75 (17)
C8—C9—C10—C15	−0.8 (2)	C9—C8—C23—O9	−0.1 (3)

### Hydrogen-bond geometry (Å, °)

**Table:**

*D*—H···*A*	*D*—H	H···*A*	*D*···*A*	*D*—H···*A*
O9—H1O9···O10	0.96 (3)	1.62 (3)	2.519 (2)	155 (3)
N6—H1N6···O1^i^	0.91 (2)	1.84 (2)	2.701 (2)	159 (2)
N6—H1N6···O7^i^	0.91 (2)	2.422 (19)	2.987 (2)	120.6 (15)
C6—H6···O3	0.878 (19)	2.38 (2)	2.706 (16)	102.3 (14)
C11—H11···F1^ii^	0.933 (19)	2.46 (2)	3.209 (2)	137.3 (18)
C16—H16A···F1	0.97 (2)	2.18 (2)	2.846 (2)	124.6 (15)
C17—H17A···O8^iii^	0.928 (19)	2.582 (19)	3.430 (3)	152.2 (14)
C17—H17B···O4^iv^	1.02 (2)	2.55 (2)	3.407 (3)	141.2 (16)
C18—H18A···O3^v^	0.940 (19)	2.47 (2)	3.241 (16)	139.2 (18)
C19—H19B···O5^vi^	0.990 (17)	2.566 (17)	3.300 (2)	131.0 (13)
C20—H20A···O7^i^	0.97 (2)	2.51 (2)	3.079 (3)	118.0 (18)
C20—H20C···O4^iv^	1.01 (3)	2.39 (3)	3.245 (3)	141.8 (19)
C21—H21B···O8^vii^	0.90 (3)	2.53 (3)	3.415 (3)	169 (2)

Symmetry codes: (i) *x*, *y*−1, *z*; (ii) −*x*+1, −*y*, −*z*+1; (iii) −*x*, −*y*+1, −*z*+1; (iv) −*x*+1, −*y*, −*z*+2; (v) *x*−1, *y*, *z*; (vi) −*x*+1, −*y*+1, −*z*+2; (vii) −*x*, −*y*+2, −*z*+1.
